# Suramin blocked hCAP18/LL-37-induced macrophage recruitment and M2 polarization to enhance the therapeutic efficacy of 1,25(OH)_2_D_3_ against hepatocellular carcinoma *in vitro* and *in vivo* mouse model

**DOI:** 10.3389/fnut.2025.1556533

**Published:** 2025-05-16

**Authors:** Huidan Zhang, Wenjing Xie, Wenliang Duan, Xueli Yuan, Yaxin Yang, Qin Chen, Yiqiang Zhu, Yuqing Chen

**Affiliations:** Jiangsu Province Key Laboratory for Molecular and Medical Biotechnology, Life Sciences College, Nanjing Normal University, Nanjing, China

**Keywords:** 1,25(OH)_2_D_3_, hCAP18/LL-37, suramin, hepatocellular carcinoma, macrophage

## Abstract

**Background:**

1,25(OH)_2_D_3_ supplementation alone does not provide sufficient benefit to hepatocellular carcinoma (HCC) patients in clinical trials. Tumor-associated macrophages (TAMs)-mediated immunosuppression is regarded as a major hurdle for the effectiveness of several treatments. Previous studies revealed that hCAP18/LL-37 was an important factor which directly suppresses the anticancer activity of 1,25(OH)_2_D_3_ on HCC cells. However, whether TAMs contribute to the limited clinical efficacy of 1,25(OH)_2_D_3_ through hCAP18/LL-37 remains unclear.

**Methods:**

Co-culture systems of HCC cells (PLC/PRF-5, Huh7) with THP-1-derived macrophages and co-xenograft mouse models were established. Anticancer activity was evaluated *in vitro* and *in vivo* mouse models using standard assays. Mechanistic investigations utilized qRT-PCR, Western blot, flow cytometry, ELISA, and immunohistochemistry. Therapeutic efficacy of 1,25(OH)_2_D_3_/suramin combination was assessed in co-xenograft and N-Nitrosodiethylamine (DEN)/Carbon tetrachloride (CCl_4_)-induced HCC models.

**Results:**

1,25(OH)_2_D_3_ (200–500 nM) promoted macrophage recruitment, M2 polarization, Akt/mTOR signal and STAT3 signal activation in HCC/macrophage co-culture systems. This effect was mediated by 1,25(OH)_2_D_3_-induced hCAP18/LL-37 overexpression, which facilitated TAM infiltration and M2 reprogramming. Suramin, a potent LL-37 inhibitor, abrogated these immunosuppressive effects by blocking LL-37 internalization, restoring M1 polarization and suppressing Akt/mTOR and STAT3 pathways. Notably, 1,25(OH)_2_D_3_/suramin combination therapy synergistically inhibited HCC proliferation, colony formation, and invasion *in vitro*. In xenograft models and DEN/CCl_4_-induced HCC models, suramin enhanced 1,25(OH)_2_D_3_’s efficacy by promoting M1 polarization, increasing intratumoral M1/M2 ratios, reducing tumor growth, and diminishing macroscopic nodules.

**Conclusion:**

The 1,25(OH)_2_D_3_-LL-37-TAM axis drives immunosuppression in HCC by modulating macrophage phenotypes. While suramin potently disrupts this axis, blocking LL-37-mediated TAMs recruitment and M2 polarization, while promoting antitumor M1 phenotype responses. These findings highlight suramin as a promising adjunct to 1,25(OH)_2_D_3_-based immunotherapy for HCC.

## 1 Introduction

Hepatocellular carcinoma (HCC) is among the five most common cancers and the third leading cause of cancer-related deaths worldwide. Scientists estimate that 1.4 million people could be diagnosed with liver cancer and 1.3 million people will die from the disease in 2040 (1). Currently, tyrosine kinase inhibitors, including two first-line therapies (sorafenib and lenvatinib) and three second-line therapies (regorafenib, cabozantinib and ramucirumab), immune checkpoint inhibitors (ICIs) and combination regimens with ICIs are approved by the FDA (Food and drug administration) for HCC systemic treatment (2, 3). Despite great breakthroughs in systemic treatments, the majority of HCC patients still obtain limited benefits (4). Therefore, there is an urgent need to develop more therapeutic strategies and overcome the mechanism of tumor microenvironment (TME) mediated drug resistance.

Tumor-associated macrophages (TAMs) are the most abundant innate immune population in the TME of HCC, mediated immunosuppression and is regarded as a major hurdle for the effectiveness of several treatments (5). In addition to liver-resident macrophages (Kupffer cells), most TAMs originate from circulating monocytes that are recruited by chemotactic signals (cytokines and chemokines) to the tumour sites, and subsequently are polarized into different types of TAMs in the TME. TAMs predominantly exhibit immunosuppressive M2-like phenotypes characterized by CD163/Arg-1 expression and IL-10 secretion, which promote angiogenesis, immunosuppression, and metastasis. Conversely, M1-like TAMs expressing iNOS/TNF-*α* exert antitumor effects. The plasticity of TAMs enables their reprogramming between these phenotypes, making them pivotal regulators of treatment efficacy (6).

Vitamin D3 (1,25-dihydroxyvitamin D3; 1,25(OH)_2_D_3_) has been widely studied for its potential role in cancer treatment.^1^ Although epidemiological studies report vitamin D deficiency in 90% of HCC patients (7), and experimental studies also show 1,25(OH)_2_D_3_ a direct anticancer role against HCC cells by inhibiting cancer cell proliferation, promoting apoptosis and reducing angiogenesis (8), clinical trials demonstrate limited efficacy of monotherapy (9, 10). Recent studies have highlighted the immunomodulatory role of 1,25(OH)_2_D_3_ in anti-tumor immunity and immunotherapy response (11). In microbial infections and some inflammatory conditions, 1,25(OH)_2_D_3_ promotes macrophage polarization toward an anti-inflammatory M2 phenotype, thereby attenuating excessive immune activation (12–14). Intriguingly, recent evidence also suggests that 1,25(OH)_2_D_3_ may similarly enhance M2 polarization, potentially facilitating metastasis in 4 T1 mammary carcinoma models (15), raising concerns regarding its immunosuppressive potential within the HCC TME.

Cathelicidin hCAP18/LL-37 is one of the most relevant vitamin D receptor (VDR) regulated target genes in human immune cells (16). This 16 kDa secreted protein undergoes proteolytic processing from its 19 kDa precursor (pre-hCAP18) to yield the bioactive 4.5 kDa LL-37 peptide (17). Substantial evidence demonstrates that macrophage-derived hCAP18/LL-37 mediates both the antimicrobial and immunomodulatory effects of 1,25(OH)_2_D_3_ during infection and inflammatory conditions (18, 19). Our recent work identified hCAP18/LL-37 as a tumor-promoting factor that stimulates HCC proliferation and growth, thereby attenuating the anticancer efficacy of 1,25(OH)_2_D_3_ in mouse models (20). However, whether hCAP18/LL-37 further enhances inhibition of 1,25(OH)_2_D_3_’s anticancer activity through TAMs remains unknown.

Here, we investigate the role of the 1,25(OH)_2_D_3_-LL-37-TAM axis in HCC using *in vitro* co-culture systems and in mouse co-xenograft models. We demonstrate that LL-37 enhances macrophage recruitment and M2 polarization, key mediators of 1,25(OH)_2_D_3_-induced immunosuppression. Additionally, an old drug (suramin) synergized with 1,25(OH)_2_D_3_ to suppress tumor growth, by reprogramming immunosuppressive M1-TAMs into antitumor effectors. These findings provide a mechanistic rationale for improving 1,25(OH)_2_D_3_-based therapy via TAM-targeted combination strategies in HCC.

## 2 Materials and methods

### 2.1 Regents

We purchased Dulbecco’s Modified Eagle’s Medium (DMEM), RPMI 1640 Medium, IFN-*γ* and IL-4 for cell culture from Gibco (Thermo Fisher Scientific, USA). Lipopolysaccharide (LPS), phorbol 12-myristate 13-acetate (PMA), phenyl methane sulfonyl fluoride (PMSF), phosphatase inhibitor, 0.1% crystal violet and BeyoClick™ EdU-555 kit were sourced from Beyotime (Nanjing, China). The hCAP18/LL-37 (AS014), VDR (sc-13133), p-Akt (Ser473) (sc-514032), Arg-1 (sc-271430), CD68 (sc-17832), CD163 (sc-20066), p-4EBP1 (Ser65) (sc-293124), MMP9 (sc-393859), iNOS (sc-7271), CD163 (sc-20066) antibodies were provided from Santa (Texas, USA). The p-mTOR (Ser2448) (CY6571) and mTOR (P42345) antibodies were purchased from Abways (Shanghai, China). The Akt (9272) and PCNA (13110) antibodies were purchased from Cell Signaling Technology (Massachusetts, USA). The p-STAT3 (AP0070), STAT3 (A1192), *β*-actin (AC026) antibodies, horseradish peroxidase (HRP)-conjugated anti-rabbit secondary antibody (AS014) and anti-mouse secondary antibody (AS003) were purchased from ABclonal (Wuhan, China). The LaminB1 (12987-1-AP) antibody was purchased from Proteintech (Wuhan, China). Brilliant Violet 605™ anti mouse CD45 (103155), PE-cy7-F4/80 (123114), Alexa Fluor® 488 anti-mouse/human CD11b (101219), APC anti-mouse CD206 (141708), and Brilliant Violet 421™ anti-mouse CD86 (105032) antibodies were sourced from Biolegend (Cal, USA). Additionally, TRIzol reagent, HiScriptIII RT SuperMix (+gDNA wiper), AceQ qPCR SYBR Green Master Mix and BCA protein assay kit were purchased from Vazyme (Nanjing, China). Suramin was purchased from MedMol (Shanghai, China). 1,25(OH)_2_D_3_ was purchased from MCE (New Jersey, USA). DAB kit was purchased from Solarbio (Beijing, China). LipoPlus transfection reagent was purhcased from Synthgene (Nanjing, China). Cytokines IL-10, TNF-*α* and human cathelicidin LL-37 ELISA kits were purchased from DUMA Technology (Shanghai, China). Calcitriol and N-Nitrosodiethylamine (DEN) were sourced from Medchemexpress (New Jersey, USA). Carbon tetrachloride (CCl_4_) was purhcased from Macklin (Shanghai, China). DNase I was purchased from Sigma (Darmstadt, Germany).

### 2.2 Cell lines, cell culture, and transfection

THP-1, PLC/PRF-5, Huh7 cells were purchased from Shanghai Institute of Cell Biology, Chinese Academy of Science. THP-1 cells were cultured in RPMI 1640 medium. Huh7, PLC/PRF-5 cells were maintained in DMEM. LL-37 peptide was synthesized by Synpeptide Inc. (Nanjing, China). pRNAT-U6.1-GFP and pcDNA3.0-Flag expression vector were kept in our laboratory. All primers were synthesized by General Biol (Anhui, China) and listed in the Supplementary Table S1. LL-37 coding sequences (GenBank accession no. 820) were amplified by qRT-PCR from mRNA of PLC/PRF-5 cells using Oligo dT_23_ that incorporated *Bam*HI and *XbaI* restriction sites, and then inserted into pcDNA3.0 via *BamH*I/*XbaI* restriction sites to generate the pcDNA3.0^LL-37^. The sequence (5’-GTCCAGAGAATCAAGGATT-3′) specifically targets the encoding LL-37 of *CAMP* gene (si-LL-37) and negative control siRNA (scrambled siRNA, si-control) were synthesized by General Biol, respectively. si-LL-37 was specifically transfected into HCC cells (PLC/PRF-5 or Huh7) using LipoPlus transfection reagent. Briefly, HCC cells were seeded in 6-well plates (2 × 10^5^cells/well) and transfected with si-LL-37 (or scrambled siRNA control) for 48 h, then co-cultured with macrophages to conduct the functional assays. LL-37-overexpressed HCC cells were constructed by transfection with pcDNA3.0^LL-37^ vectors using LipoPlus transfection reagent. Conditioned medium (CM) was obtained by collecting supernatants after filtration through 0.22 μm pore size membranes. CM was mixed with fresh RPMI 1640 or DMEM medium at a ratio of 1:1 (CM/fresh medium).

### 2.3 M1 and M2 macrophage polarization induction

M1-type and M2-type macrophages were induced from THP-1 as described previously (21). Briefly, THP-1 cells were differentiated into an intermediate stage M0 by stimulation with 100 ng/mL PMA for 24 h. For M1 macrophage polarization, 100 ng/mL LPS and 20 ng/mL IFN-*γ* were added to M0 macrophages for 48 h. For macrophage M2 polarization, 20 ng/mL IL-4 was added to M0 macrophages for 48 h.

### 2.4 Transwell recruitment assay and invasion assay chemotaxis

A co-culture system of macrophages and HCC cells (PLC/PRF-5, Huh7) was established using 24-well transwell plates with transwell chambers with 8-μm poresize (Biofil, Guangzhou, China). HCC cells or macrophages were seeded in the bottom chamber, while macrophages or HCC cells were cultured in the upper chamber. For transwell recruitment assay, 1 × 10^5^ M0 macrophages were resuspended in 200 μL of 1% FBS RPMI 1640 medium and plated in the upper chamber of a 24-well plate. 1 × 10^5^ HCC cells were placed the lower chamber. For invasion assay, the transwell chambers were coated with 100 μL of 1:8 diluted Matrigel and incubated at 37°C for 4 h. The HCC cells (0.5–1 × 10^5^ cells) were cultured in the upper chamber and macrophages (0.5–1 × 10^5^ cells) were cultured in the bottom chamber. After different treatments for 48 h, the cells in the upper chamber were fixed with 4% paraformaldehyde (PFA) for 15 min and then stained with 0.1% crystal violet for 20 min. The non-migrated cells in the upper chamber were removed with a cotton swab, and migratory cells were observed with a microscopy. Four random visual fields in each well were visualized under a microscope for cell counting. After elution with 33% glacial acetic acid, cells were detected at 570 nm. Each independent experiment was repeated four times.

### 2.5 Western blot assay

Protein lysates from isolated tissues or cultured cells were extracted using RIPA buffer containing PMSF and phosphatase inhibitors. The protein concentration was determined by BCA protein assay kits. Protein (20 μg) was separated by SDS-PAGE, and then transfered to a PVDF membrane (Millipore, Darmstadt, Germany). After blocking, the membranes were incubated with primary antibodies specific to hCAP18/LL-37, VDR, p-Akt (Ser473), Arg-1, CD68, CD163, p-4EBP1 (Ser65), MMP9, iNOS, p-mTOR (Ser2448), mTOR Akt, *β*-actin, p-STAT3 (Tyr705), STAT3 or LaminB1 overnight at 4°C. Subsequently, the PVDF membranes were incubated with horseradish peroxidase (HRP)-conjugated anti-rabbit secondary antibody or anti-mouse secondary antibody for 1 h at room temperature. Membranes were incubated with ECL substrate and the targeted proteins were visualized with a chemiluminescence imaging system (Tanon, China).

### 2.6 Quantitative real-time PCR (qRT-PCR)

Total RNA was extracted from cells using TRIzol reagent and cDNA was synthesized using HiScriptIII RT SuperMix with Oligo (dT)_23_/random hexamer primers. qPCR was performed on Thermal Cycler 96-well Real-Time PCR Detection System (Thermo scientific, MA, USA) with AceQ qPCR SYBR Green Master Mix. Gene expression was normalized to *β*-actin and calculated using the 2^−ΔΔCt^ method. All primers were synthesized by General Biol (Anhui, China) and listed in the Supplementary Table S2.

### 2.7 EdU incorporation assay

Cell proliferation was assessed using the BeyoClick™ EdU-555 Kit following the manufacturer’s protocol. Briefly, after different treatments, cells were incubated with 10 μM EdU for 2 h, fixed with 4% PFA for 15 min and permeabilized with 0.2% Triton X-100 for 15 min. Then Cells were incubated with Click Addictive Buffer for 30 min at room temperature (protected from light). After washing with PBS, cells were stained with 4′,6-diamidino-2-phenylindole (DAPI) at a concentration of 10 μg/mL for 15 min to visualize nuclei. More than five random fields per well were quantified as a percentage of EdU-positive cells.

### 2.8 Colony formation assay

Cells were seeded in 12-well plates and cultured in either standard DEME (control) or conditioned medium (CM:DMEM = 1:1). After 10 days of culture, cells were fixed with 4% PFA for 15 min and then stained with 0.1% crystal violet for 20 min. Colonies were photographed and counted. Each independent experiment was repeated four times.

### 2.9 Confocal laser scanning microscopy

PLC/PRF-5 and macrophages were incubated with FITC-labeled LL-37 (1 μM), suramin (5 μM) alone or combination (1 μM LL-37 + 5 μM suramin) for 30 min in the dark. After washing, the cells were incubated with DAPI (5 μM) for 15 min in the dark, followed by washing with PBS. Cells were observed using a Nikon Ti-E-A1R confocal microscope (Tokyo, Japan).

### 2.10 Subcutaneous xenograft tumor model

Male Balb/c nude mice (4–6 weeks old, 18.0 ± 2.0 g; GemPharmatech, China) were housed under SPF conditions. All procedures were approved by Nanjing Normal University’s Ethics Committee (IACUC-20220218). Two models were established: PLC/PRF-5 cells (6 × 10^6^) were injected subcutaneously (*n* = 10) to establish HCC xenograft model, and PLC/PRF-5 (6 × 10^6^) plus M0 macrophages (1.5 × 10^6^) were injected subcutaneously to establish HCC/macrophage co-xenograft model (*n* = 40). At tumor volumes of 50–100 mm^3^, co-xenograft mice were randomized into four treatment groups (*n* = 8/group): (1) PBS group, (2) 1,25(OH)_2_D_3_ group (a dose of 0.5 μg/kg/per day), (3) suramin group (10 mg/kg twice a week), and (4) combination therapy 1,25(OH)_2_D_3_/suramin group. Tumor volume was calculated using the formula V = ab^2^/2, where “a” and “b” are tumor dimensions at the longest and widest points, respectively. The mice were sacrificed by isoflurane/cervical dislocation. Tumors were excised, weighed, and the inhibition rate calculated as: (TW_control_ − TW_Treatment_) /TW_control_ × 100%.

### 2.11 Immunofluorescence and immunohistochemistry microscopy observation

For IF assay, cells platedon glass slides were fixed (4% PFA), permeated (1% Triton X-100), and blocked (5% BSA, 37°C, 1 h). Then the slides were incubated with indicated promary antibodies at 4°C overnight, followed by incubating with fluorescence-conjugated secondary antibodies at room temperature for 1 h. Slides were stained with DAPI for 15 min to visualize nuclear DNA. After washing, cells were imaged with a Ti-E-A1R confocal laser microscope. For IHC assay, paraffin-embedded tissue sections were prepared as previously described (20). After repairing antigen with boiled Ethylene Diamine Tetraacetic Acid (EDTA) solution and quenching endogenous peroxidase with 3% H_2_O_2_, the sections were incubated by primary antibodies CD163, hCAP18/LL-37, Arg-1, iNOS, PCNA at 4°C overnight. After washing, the sections were incubated with HRP-conjugated goat anti-rabbit IgG or HRP-conjugated goat anti-mouse IgG secondary antibody for 1 h at room temperature. Finally, the sections were visualized with DAB kit, counterstained with hematoxylin, dehydrated and examined by an Olympus IX51 fluorescence microscope. Approximately 4–6 fields were randomly selected for each sample. Data were collected from 4 to 6 independent experiments.

### 2.12 Enzyme-linked immunosorbent assay (ELISA)

Cytokines (IL-10 and TNF-*α*) levels in the culture supernatant was quantified using the commercial ELISA kits. Mouse blood was collected and centrifuged at 1500 rpm for 10 min to collect serum. Serum level of hCAP18/LL-37 was determined by human cathelicidin ELISA kit. All assays followed manufacturers’ protocols.

### 2.13 DEN/CCl_4_- induced HCC mouse model

Male C57BL/6 mice (3-weeks-old) were purchased from GemPharmatech Co. Ltd. and maintained under SPF conditions. Animal procedures and experimental methods were approved by the Ethics Committee of Nanjing Normal University (IACUC-2024210). To establish a HCC model, mice received intraperitoneal injections of DEN at 50 mg/kg biweekly for 4 weeks, followed by CCl_4_ dissolved in olive oil (1:4) administered intraperitoneally biweekly for an additional 16 weeks. After the successful construction of the model, the mice were randomly divided into 4 groups (*n* = 8 per group): (1) PBS group, (2) 1,25(OH)_2_D_3_ group (a dose of 0.5 μg/kg per day), (3) suramin group (10 mg/kg twice a week), and (4) combination therapy (1,25(OH)_2_D_3_/suramin) group. All treatments were initiated post-model confirmation and continued for 3 weeks. The mice were sacrificed by isoflurane/cervical dislocation, livers were excised, weighed. and macroscopic nodules (≥1 mm) counted for flow cytometry analysis.

### 2.14 Flow cytometry analysis

Tissues were enzymatically dissociated into single-cell suspensions using Hank’s Balanced Salt Solution (HBSS) containing 1 mg/mL collagenase and 50 μg/mL DNase I at 37°C for 30 min. After erythrocyte lysis and 70-μm filtration, cells were resuspended in PBS containing 3% FBS for Fc receptor blocking (10 min). The following primary antibodies were incubated for 30 min: Brilliant Violet 605™ anti mouse CD45, PE-cy7-F4/80, Alexa Fluor® 488 anti-mouse/human CD11b, APC anti-mouse CD206, or Brilliant Violet 421™ anti-mouse CD86 antibodies. Additionally, cells from xenograft tumor were stained with mouse anti-human CD163 antibody at 4°C overnight. After washing with PBS, cells were incubated with Alexa Fluor® 488-conjugated mouse IgG secondary antibody at 4°C for 1 h. Flow cytometry was performed using a Cytek® NL-CLC full spectrum flow cytometry (CYTEK, CA, USA). Data were analyzed using FlowJo 10.8.1 software (TreeStar, Inc.).

### 2.15 Statistical analysis

Experiments were independently repeated 4–6 times with biological replicates. Data are expressed as means ± SEM. Two-tailed Student’s t-test for pairwise comparisons and one-way ANOVA followed by Tukey’s test for multiple group comparisons were used to determine the significance of differences. All statistical analyses were performed using GraphPad Prism 6 (La Jolla, CA). *p*-value < 0.05 was considered statistically significant.

## 3 Results

### 3.1 1,25(OH)_2_D_3_ enhances macrophage recruitment/M2 polarization associated with LL-37 level

**Figure 1:**
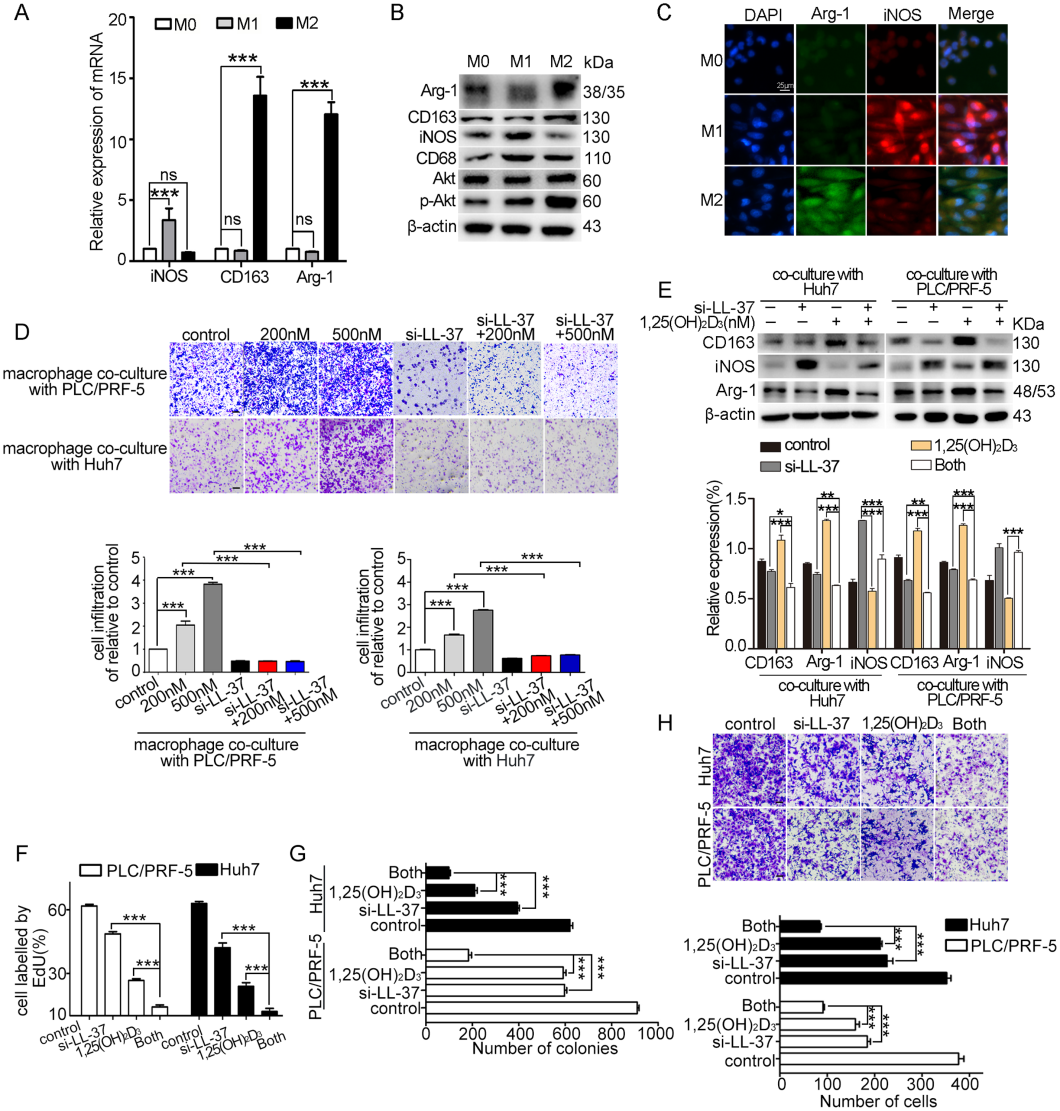
Effects of 1,25(OH)_2_D_3_ and si-LL-37 on macrophage recruitment and polarization in HCC/macrophage co-culture systems. **(A)** THP-1 derived M1-type and M2-type macrophages were identified using the M1 markers (iNOS) and M2 markers (CD163, Arg-1) by qRT-PCR, respectively. **(B)** Western blot analysis of M1 markers and M2 markers in macrophages. **(C)** Immunofluorescence staining of iNOS and Arg-1 in M0, M1-type and M2-type macrophages. Scale bars, 20 μm. **(D)** PLC/PRF-5 and Huh7 cells were transfected with si-LL-37 for 8 h to obtain LL-37-knockdowned cells (PLC/PRF-5^LL-37/low^ or Huh7^LL-37/low^). These modified HCC cells were cultured in the bottom chamber. Then transwell recruitment assay was conducted to assess the macrophage migration to HCC cells after 1,25(OH)_2_D_3_ treatment when co-cultured with HCC cells or modified HCC cells (si-LL-37 transfection). Scale bars, 50 μm. **(E)** Western blot assay was conducted to detect the expression of M1-type and M2-type markers in macrophages with or without 1,25(OH)_2_D_3_. EdU assay **(F)**, colony formation assay **(G)** and invasion assay **(H)** were conducted to detect proliferation, clonogenesis and invasion of PLC/PRF-5 and Huh7 cells in HCC/M0 co-culture model after 1,25(OH)_2_D_3_ mono-treatment or combination with si-LL-37. Scale bars, 50 μm. Data are represented as the mean ± SEM of 4–6 different experiments. *p < 0.05, **p < 0.01, ***p < 0.001.

THP-1-derived M1-type and M2-type macrophages were identified by the classical M1-type markers (iNOS) and M2-type markers (CD163, Arg-1), respectively (Figures 1A–C). Subsequently, we established a HCC/macrophage co-culture system to investigate the effect of 1,25(OH)_2_D_3_ on macrophage recruitment and polarization. THP-1-derived M0 macrophages were cultured in upper inserts, while HCC cells (PLC/PRF-5/Huh7) were seeded in lower chambers. Transwell recruitment assays revealed that 1,25(OH)_2_D_3_ treatment (200–500 nM) significantly enhanced macrophage recruitment to HCC cells (*p* < 0.001) (Figure 1D). Notably, siRNA-mediated LL-37 knockdown (si-LL-37) completely abolished this chemotactic response (*p* < 0.001), confirming LL-37 as the critical mediator. Furthermore, 1,25(OH)_2_D_3_ treatment significantly upregulated canonical M2 polarization markers (Arg-1 and CD163, *p* < 0.001) in co-cultured macrophages (Figure 1E), suggesting an increase of M2 polarization induced by 1,25(OH)_2_D_3_. However, this pro-tumorigenic polarization was entirely negated by si-LL-37 pretreatment, confirming the essential role of LL-37 in mediating 1,25(OH)_2_D_3_-dependent macrophage reprogramming. Functional analyses demonstrated that, combined treatment with si-LL-37 significantly enhanced 1,25(OH)_2_D_3_-induced the suppression of HCC cell proliferation, colony formation, and invasion through Matrigel in co-culture models (*p* < 0.001) (Figures 1F–H). These results demonstrate that 1,25(OH)_2_D_3_ enhances macrophage recruitment and M2 polarization, as well as limits the anticancer activity of 1,25(OH)_2_D_3_ in HCC/macrophage co-culture model, while LL-37 as the critical mediator during these processes.

### 3.2 LL-37 stimulates macrophages recruitment and M2 polarization *in vitro*

**Figure 2:**
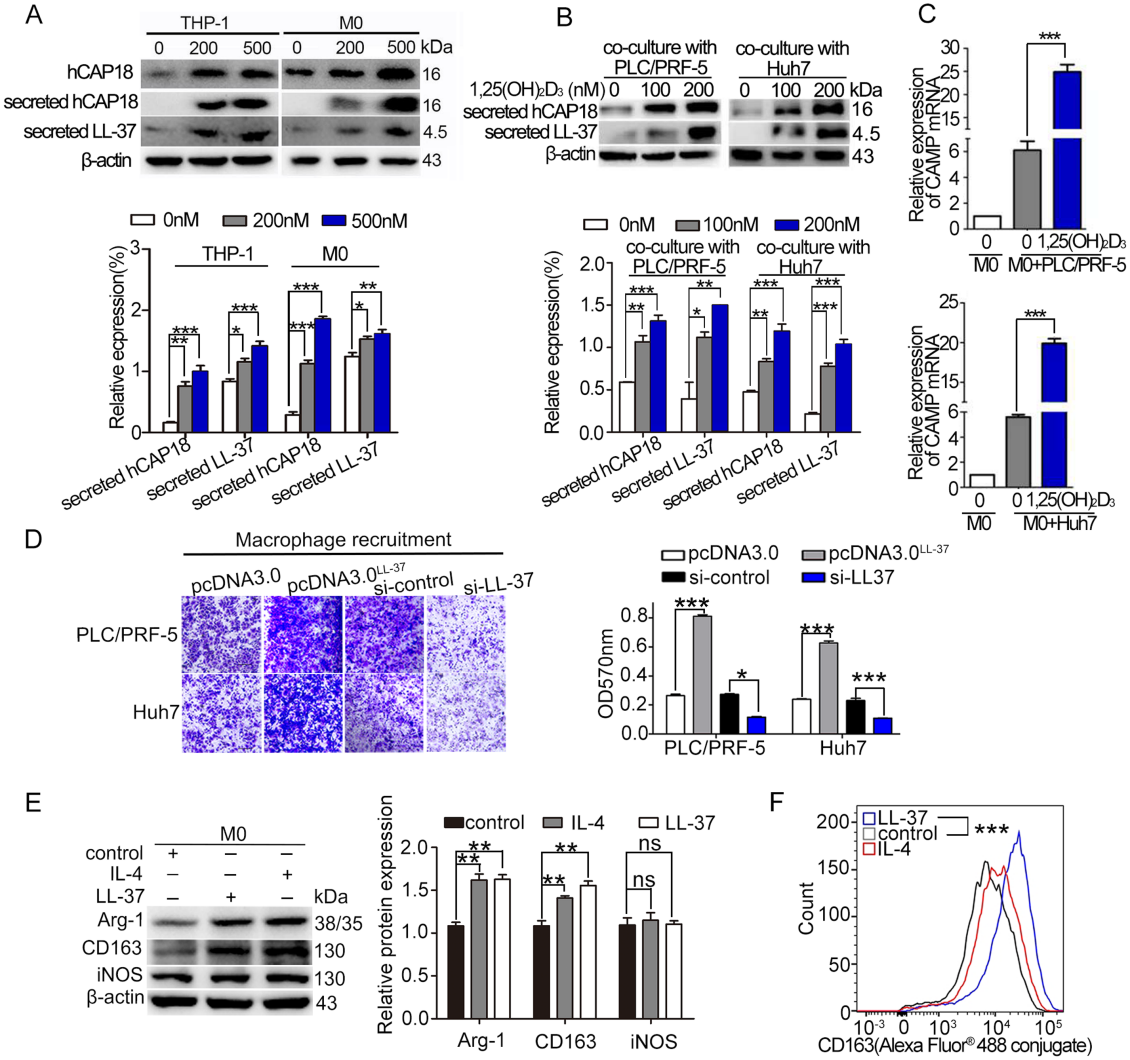
1,25(OH)_2_D_3_ modulates hCAP18/LL-37 expression and macrophage recruitment/polarization. **(A)** Western blot analysis of the levels of intracellular hCAP18 (cell lysates) and secreted hCAP18/LL-37 (media) hCAP18/LL-37 in THP-1-derived macrophages treated with 1,25(OH)_2_D_3_ for 48 h. **(B)** Secreted hCAP18/LL-37 protein levels were detected in HCC/macrophage co-cultures after 1,25(OH)_2_D_3_ treated for 48 h. **(C)** *CAMP* mRNA level in co-cultured macrophages were quantified by qRT-PCR after 1,25(OH)_2_D_3_ (200 nM) treated for 48 h. **(D)** Transwell recruitment assays were performed in transwell chambers. PLC/PRF-5 and Huh7 cells were transfected with pcDNA^LL-37^ or si-LL-37 for 8 h to obtain LL-37-overexpressed cells (PLC/PRF-5^LL-37/high^, Huh7^LL-37/high^) or LL-37-knockdowned cells (PLC/PRF-5^LL-37/low^ or Huh7^LL-37/low^). These modified HCC cells were cultured in the bottom chamber. After 48 h, macrophages (in the upper chamber) were stained with crystal violet and detected at 570 nm. Scale bars, 50 μm. **(E)** Western blot analysis of CD163, Arg-1 and iNOS in M0 macrophages treated with LL-37 (2 μM) or IL-4 (20 ng/mL) for 48 h. **(F)** Flow cytometric quantification of CD163 level in macrophages following LL-37 treatment. Data are mean±SEM (*n* = 4–6). ns, no significance, *p < 0.05, **p < 0.01, ***p < 0.001.

**Figure 3:**
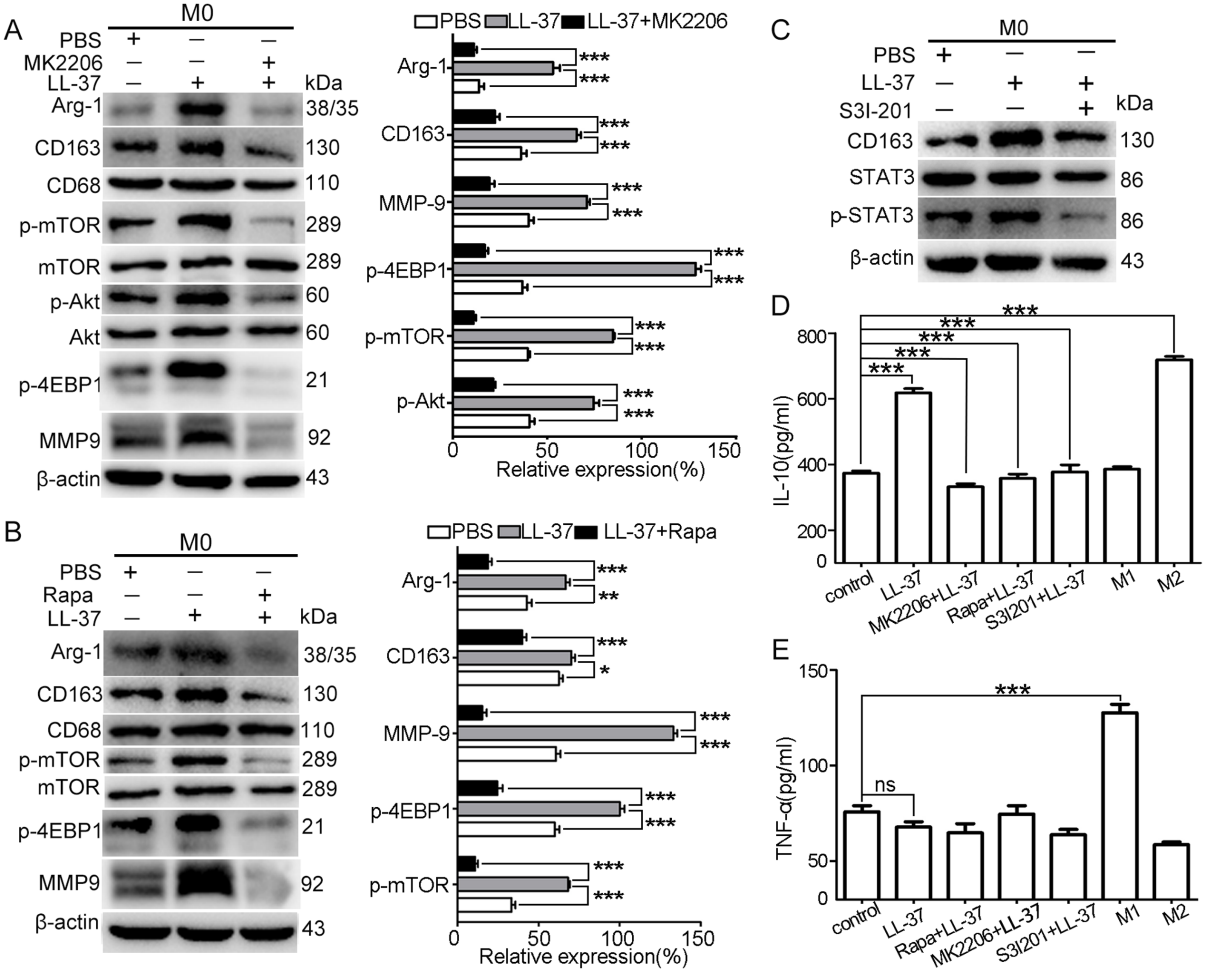
Akt/mTOR and STAT3 signals during LL-37-stimulated macrophage M2 polarization. After treatment of M0 macrophages with LL-37 (2 μM) for 48 h, western blot analysis of M2 markers (Arg-1, CD163, CD68) and phosphorylation levels (p-mTOR, p-Akt, p-4EBP1) in LL-37-treated M0 macrophages with pathway-specific inhibitors: **(A)** p-Akt inhibitor MK2206, **(B)** p-mTOR inhibitor Rapa, and **(C)** p-STAT3 inhibitor S3I201. ELISA quantification of anti-inflammatory (IL-10) and pro-inflammatory (TNF-*α*) cytokines secreted by LL-37-stimulated macrophages. Data are mean ± SEM of 4–6 different experiments. ns, no significance, *p < 0.05, **p < 0.01, ***p < 0.001.

To elucidate the mechanisms by which si-LL-37 inhibited 1,25(OH)_2_D_3_-mediated macrophage recruitment and M2 polarization, we first investigated the impact of 1,25(OH)_2_D_3_ treatment on hCAP18/LL-37 levels in HCC/macrophage co-culture systems. Here, we confirmed that 1,25(OH)_2_D_3_ (200 nM, 24 h) significantly upregulated secreted hCAP18/LL-37 protein levels in both THP-1 and M0 macrophages (*p* < 0.05) (Figure 2A). In HCC/macrophage co-cultures, 1,25(OH)_2_D_3_ treatment resulted in ~ 4 fold induction of secreted LL-37 protein (Figure 2B) and 3 ~ 4 fold increase in *CAMP* mRNA (*p* < 0.001) (Figure 2C). Mechanistic investigations revealed that 1,25(OH)_2_D_3_ induced nuclear translocation of VDR in both PLC/PRF-5 and macrophages (Figure S1A). ChIP-PCR assay and dual luciferase reporter gene assay demonstrated direct VDR binding to the vitamin D response element (VDRE) in the *CAMP* promoter, driving transcriptional activation in both cell types (Supplementary Figures S1B,C). These findings establish VDR-mediated transcriptional regulation of hCAP18/LL-37 in HCC cells and macrophages.

Subsequently, we investigated the effect of LL-37 on macrophage recruitment and polarization. M0 macrophages were cultured in upper inserts, while HCC cells (PLC/PRF-5/Huh7 wild-type or their LL-37-overexpressing/knockdown derivatives) were seeded in lower chambers. Chemotaxis assay revealed that LL-37-overexpressing HCC cells (PLC/PRF-5^LL-37/high^ and Huh7^LL-37/high^) significantly enhanced macrophage recruitment compared to control (*p* < 0.001) (Figure 2D), whereas LL-37-knockdowned HCC cells (PLC/PRF-5^LL-37/low^ and Huh7^LL-37/low^) showed markedly reduced recruitment capacity (*p* < 0.05). The effect of LL-37 on M2 polarization was also detected by M2-type markers (Arg-1 and CD163) levels using exogenous addition of LL-37. Similar to the IL-4 induction, LL-37 significantly increased the levels of M2 markers in macrophages (*p* < 0.01) (Figure 2E). Flow cytometry further demonstrated that CD163 (Alexa Fluor® 488 conjugate) fluorescence was increased approximately double higher after LL-37 treatment (*p* < 0.001) (Figure 2F). Together, these results suggest that LL-37 stimulates the recruitment and M2 polarization of THP-1 derived macrophages *in vitro*.

### 3.3 Akt/mTOR and STAT3 signals mediate LL-37-stimulated M2 macrophage polarization

To elucidate the signal mechanisms driving LL-37-mediated M2 polarization, we investigated the regulatory role of the Akt/mTOR and STAT3 signaling pathways. Exogenous LL-37 treatment (100 ng/mL, 48 h) induced significant upregulation of M2 markers (CD163/Arg-1) and MMP9, accompanied by marked phosphorylation of Akt (p-Akt), mTOR (p-mTOR), and 4E-BP1 (p-4E-BP1) in macrophages (*p* < 0.001) (Figure 3A). Using Akt inhibitor MK2206 or mTOR inhibitor Rapamycin (Rapa) effectively reversed these LL-37-mediated enhancements (Figures 3A,B). Meanwhile, p-STAT3 level was increased after LL-37 treatment, while blockade of p-STAT3 by S3I201 obviously decreased the LL-37-induced increase of p-STAT3 level (Figure 3C). Functional validation through ELISA revealed that LL-37 stimulation significantly increased IL-10 secretion (M2-specific cytokine; *p* < 0.001), while MK2206, Rapa or S3I201 completely abolished LL-37-induced IL-10 upregulation (*p* < 0.001) (Figures 3D,E). Notably, LL-37 treatment does not significantly alter TNF-*α* (M1-specific cytokine) production in macrophages. Together, these findings demonstrate that both Akt/mTOR and STAT3 pathways are involved in LL-37-stimulated macrophage M2 polarization.

### 3.4 Suramin reverses the effect of 1,25(OH)_2_D_3_ on macrophages

**Figure 4:**
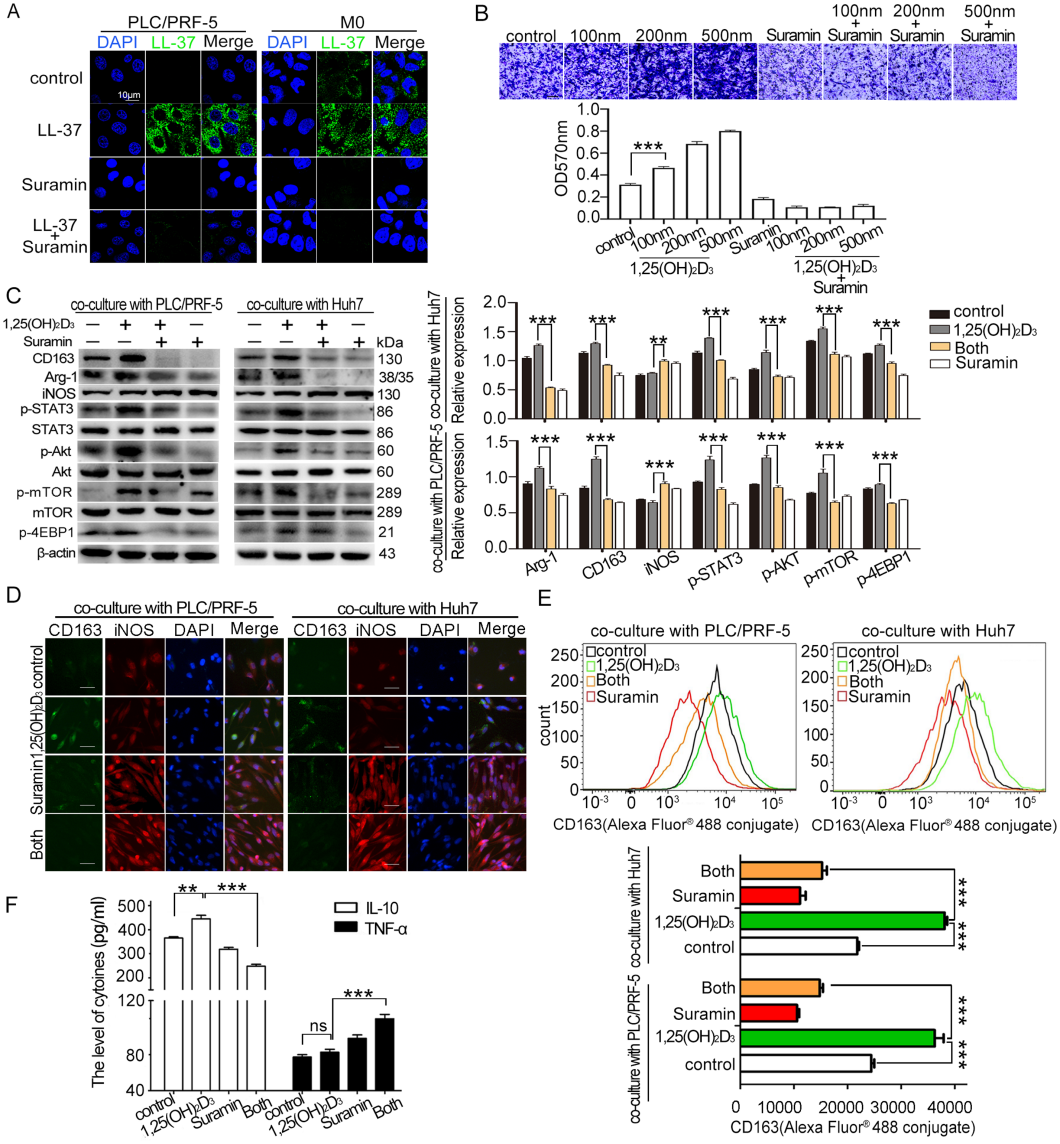
Effect of suramin on 1,25(OH)_2_D_3_-induced recruitment and M2 polariation. **(A)** Confocal microscopy analysis of LL-37 distribution (FITC-green) in PLC/PRF-5/macrophage co-cultures treated with and FITC-LL-37 (1 μM) and suramin (5 μM). DAPI (blue) marks nuclei. Scale bar, 10 μm. **(B)** Transwell recruitment assay quantifying THP-1 monocyte (upper layer) recruitment to HCC cells (bottom layer) **(C)** Western blot analysis of M1/M2 markers (iNOS/CD163), Akt/mTOR (p-Akt, p-mTOR, p4EBP1), and p-STAT3 phosphorylation in co-cultured macrophages after 1,25(OH)_2_D_3_ (200 nM) mono-treatment or combination with suramin (5 μM). **(D)** After incubation with anti-CD163 antibody, anti-iNOS antibody and Alexa Fluor® 488-conjugated mouse IgG, CD163 and iNOS levels in macrophages was observed by immunofluorescence assay. Scale bars, 20 μm. **(E)** After incubation with Alexa Fluor® 488-conjugated anti-CD163, flow cytometric quantification of CD163 level in macrophages. **(F)** ELISA detection of IL-10 and TNF-α in co-culture supernatants. Levels of IL-10 and TNF-α in culture supernatant of HCC/macrophage co-cultures (*n* = 4 samples per group). Data are mean±SEM (*n* = 4–6). ns, no significance, **p < 0.01, ***p < 0.001.

Suramin, an old antiparasitic agent with established LL-37-binding capacity (22), was evaluated for its capacity to reverse 1,25(OH)_2_D_3_-induced macrophage recruitment and M2 polarization in HCC/macrophage co-culture model. Results showed that suramin treatment (5 μM) completely inhibited the membrane binding and internalization of LL-37 in both PLC/PRF-5 cells and macrophages (Figure 4A). Functional analyses revealed that suramin treatment abolished 1,25(OH)_2_D_3_-induced macrophage recruitment (*p* < 0.001) (Figure 4B). Suramin decreased 1,25(OH)_2_D_3_-upregulated M2 markers (Arg-1, CD163) while increased M1 marker iNOS level when co-cultured with HCC cells (PLC/PRF-5 or Huh7) (*p* < 0.001) (Figures 4C,D). During the process, suramin potently suppressed 1,25(OH)_2_D_3_-activated Akt/mTOR signaling, as judged by the phosphorylation inhibition of Akt, mTOR, and 4EBP1 (*p* < 0.001) (Figure 4C). Flow cytometric validation confirmed suramin’s capacity to reverse 1,25(OH)_2_D_3_-mediated M2 polarization, showing 150% reduction in CD163^+^ macrophage populations compared to 1,25(OH)_2_D_3_-treated control (*p* < 0.001) (Figure 4E). Secreted cytokines analysis further demonstrated that suramin significantly decreased the 1,25(OH)_2_D_3_-upregulated IL-10 (M2 type) release, while increased 1,25(OH)_2_D_3_-downregulated TNF-*α* (M1 type) release from macrophages (*p* < 0.001) (Figure 4F). Together, these data demonstrate that suramin effectively reverses 1,25(OH)_2_D_3_-induced macrophage recruitment and M2 polarization in HCC/macrophage co-culture model *in vitro*.

### 3.5 Suramin enhances the anticancer activity of 1,25(OH)_2_D_3_ in HCC/macrophage co-culture model

**Figure 5:**
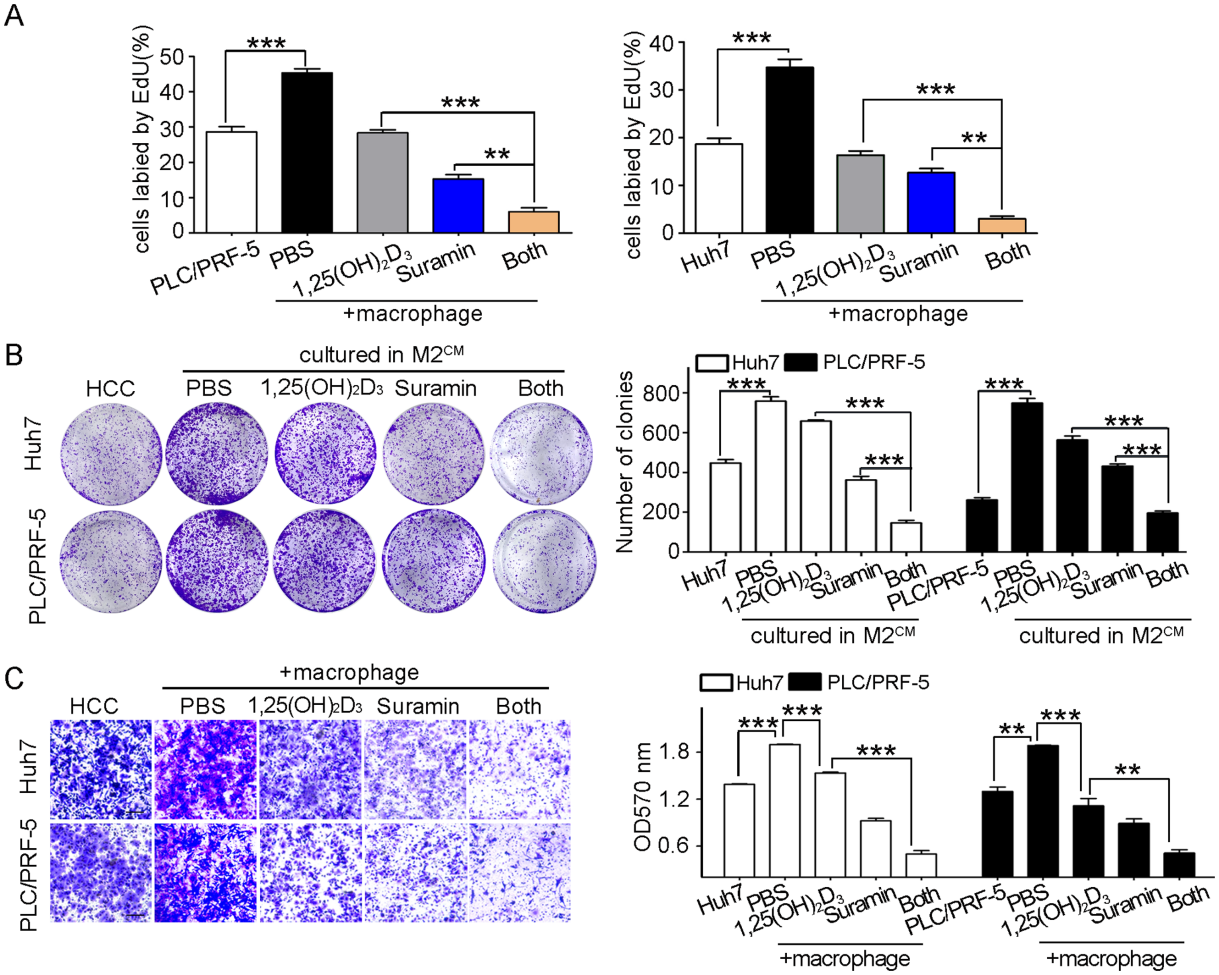
Suramin promotes the anticancer activity of 1,25(OH)_2_D_3_ in HCC/macrophage co-cultures. PLC/PRF-5 or Huh7 cells (bottom layer) were co-cultured with M0 macrophages (upper layer) and then treated with 1,25(OH)_2_D_3_ (200 nM) and/or suramin (5 μM). **(A)** EdU assays detected the proliferation of PLC/PRF-5 and Huh7 cells. **(B)** Colony formation assay was conducted to detect the growth and survival of PLC/PRF-5 and Huh7 cells in M2^CM^. **(C)** Transwell assays detected the invasion of PLC/PRF-5 and Huh7 cells when co-culture with M0 macrophages. Scale bars, 50 μm. Data are represented as the mean±SEM of 4–6 different experiments. **p < 0.01, ***p < 0.001.

To evaluate suramin’s synergistic effects on 1,25(OH)_2_D_3_-mediated anticancer activity, we performed proliferation, clonogenic, and invasion assays in HCC/macrophage co-culture systems. Co-culture with M0 macrophages (upper layer) significantly enhanced the proliferation of HCC cells (PLC/PRF-5 and Huh7, bottom layer) by 40 ~ 50% compared to HCC monoculture (*p* < 0.001) (Figure 5A), as evidenced by an increase in EdU-positive cells. Notably, 1,25(OH)_2_D_3_ mono-treatment inhibited the proliferation by 40 ~ 50%, while suramin co-treatment synergistically reduced proliferation to < 5% (*p* < 0.001). In M2-type macrophage-derived conditioned medium (M2^CM^) assays, suramin further enhanced 1,25(OH)_2_D_3_’s inhibitory effects on colony formation (*p* < 0.001) (Figure 5B), demonstrating enhanced suppression of malignant potential. While M0 macrophages (bottom chamber) promoted HCC cell (upper chamber) invasion by ~ 40% (*p* < 0.001), 1,25(OH)_2_D_3_ reduced this invasion by 20% to Huh7 and 45% to PLC/PRF-5, respectively. Importantly, suramin combination treatment achieved 70% invasion inhibition compared to 1,25(OH)_2_D_3_ alone (*p* < 0.001) (Figure 5C). These findings confirm that suramin potently enhances 1,25(OH)_2_D_3_’s anticancer efficacy through synergistic suppression of proliferation, clonogenicity, and invasion in HCC/macrophage microenvironments.

### 3.6 Suramin enhances the antitumor effect of 1,25(OH)_2_D_3_ in HCC/macrophage co-xenografts

**Figure 6:**
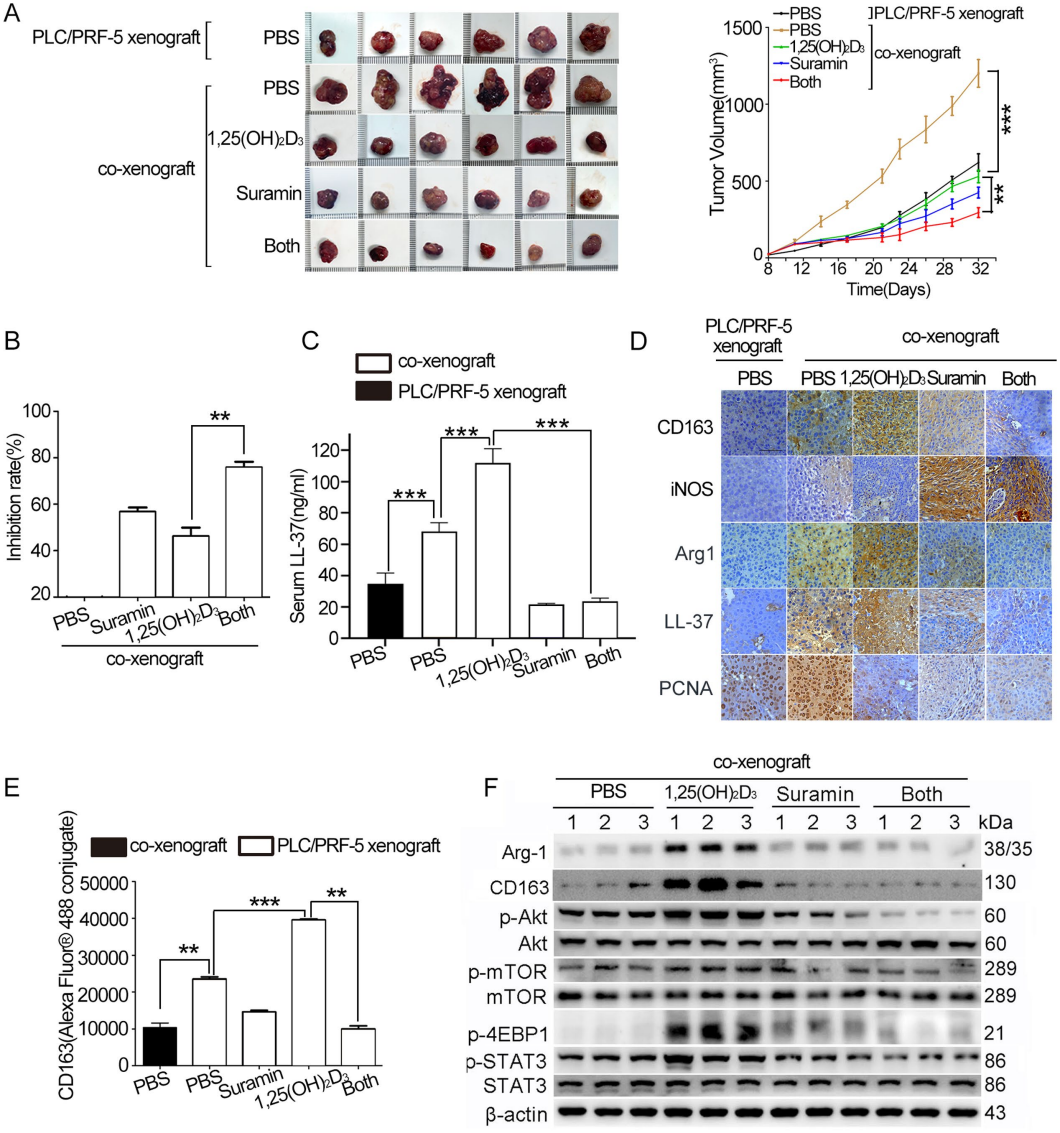
Suramin enhances the antitumor efficacy of 1,25(OH)_2_D_3_ in PLC/PRF-5/macrophage co-xenografts. Four to 6 weeks-old nude mice were subcutaneously injected with PLC/PRF-5 cells (6 × 10^6^) or a mixture of PLC/PRF-5 cells (6 × 10^6^) plus M0 macrophages (1.5 × 10^6^). About 4 weeks later, the mice were euthanized and tumor tissue dissected for analysis. Tumor volume **(A)** was determined every 4 days and tumor growth curves were illustrated. Tumor images of each group at the end of treatment were shown. **(B)** The tumor growth inhibition rate was calculated from tumor weights. **(C)** Serum hCAP18/LL-37 levels quantified by ELISA assay. **(D)** Paraffin sections were prepared for IHC staining using anti-CD163, anti-Arg-1, anti-hCAP18/LL-37, and anti-PCNA antibodies, respectively. Representative images from each group are shown. Scale bar, 50 μm. **(E)** After incubation with anti-CD163 and secondary antibody (Alexa Fluor® 488-conjugated mouse IgG), flow cytometry was performed to detect the CD163 level. **(F)** Tumor tissues were disassociated to collect cells. Cells were lysed with RIPA lysis buffer, and the supernatants were collected for western blot analysis using indicated antibodies. Data are mean±SEM. **p < 0.01, ***p < 0.001.

We established subcutaneous PLC/PRF-5 xenografts and HCC/macrophage co-xenografts (Balb/c nude mice injected with PLC/PRF-5 or PLC/PRF-5 + M0 cells). As shown in Supplementary Figure S1D, body weight remained stable across groups. Co-injection with M0 macrophages accelerated tumor growth, yielding double larger volumes (1,208 ± 90 mm^3^ vs. 606 ± 65 mm^3^) compared to monoculture controls (*p* < 0.001) (Figure 6A). While 1,25(OH)_2_D_3_ and suramin mono-treatment inhibited co-xenograft growth by 44 and 56% (*p* < 0.01), respectively, their combination achieved synergistic suppression (75% inhibition vs. mono-treatment) (*p* < 0.01) (Figure 6B). ELISA revealed that co-xenografted mice exhibited 2.0-fold elevated serum hCAP18/LL-37 levels versus PLC/PRF-5 control (*p* < 0.001). Although 1,25(OH)_2_D_3_ further increased these levels by 40%, suramin monotherapy or combination treatment reduced them by 70% (*p* < 0.001) (Figure 6C). IHC analysis demonstrated that 1,25(OH)_2_D_3_ induced hCAP18/LL-37 level upregulation and M2 polarization, evidenced by CD163^+^/Arg-1^+^/iNOS^−^ staining (Figure 6D). While suramin abrogated this effect, restoring M1 phenotype (iNOS^+^/CD163^−^) in co-treated tumors (*p* < 0.001). Flow cytometry revealed that 1,25(OH)_2_D_3_ treatment significantly increased CD163 level in co-xenograft tumor (*p* < 0.001). However, co-treatment with suramin obviously reduced the CD163 level (*p* < 0.01) (Figure 6E). Western blot analysis showed that 1,25(OH)_2_D_3_ upregulated p-Akt, p-mTOR, p-4EBP1 and p-STAT3. Suramin treatment completely reversed these phosphorylation activations in both mono- and combination therapies (Figure 6F). These findings demonstrate that suramin enhances the antitumor activity of 1,25(OH)_2_D_3_ through reprogramming M1 macrophage polarization, and suppressing oncogenic Akt/mTOR and STAT3 signals.

### 3.7 Suramin enhances the antitumor effect of 1,25(OH)_2_D_3_ in DEN/CCl_4_-induced HCC mouse model

**Figure 7:**
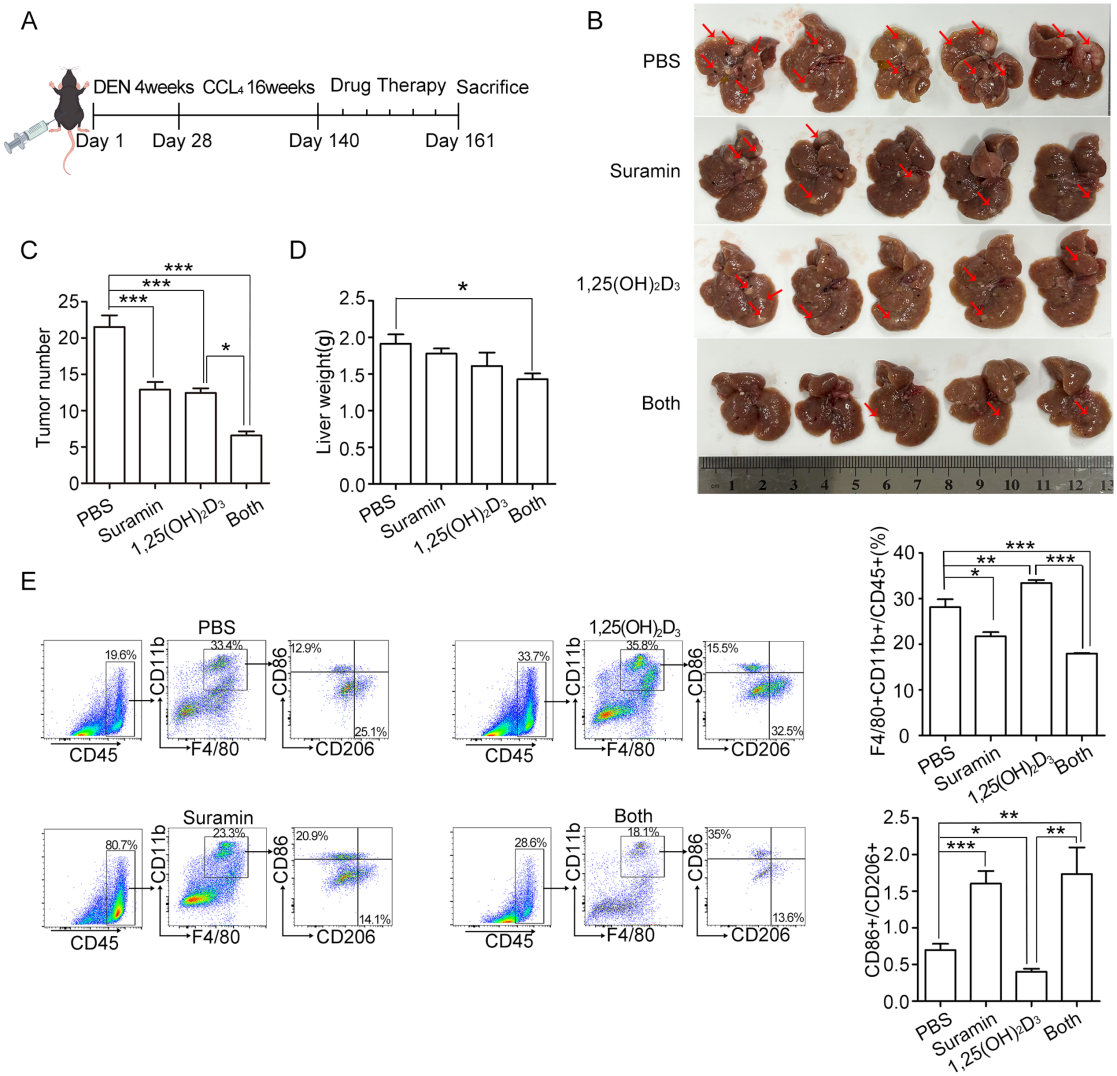
Suramin enhances the antitumor effect of 1,25(OH)_2_D_3_ in DEN/CCl₄-induced HCC model. **(A)** the schedule of DEN/CCl_4_-induced HCC modeling and administration of primary liver cancer mice. About 20 weeks later, the mice were randomly divided into 4 groups (*n* = 8 per group): (1) PBS group, (2) 1,25(OH)_2_D_3_ group (a dose of 0.5 μg/kg per day), (3) suramin group (10 mg/kg twice a week), and (4) 1,25(OH)_2_D_3_ plus suramin group. All treatments were initiated post-model confirmation and continued for 3 weeks. Mice were euthanized and tumor tissue dissected for analysis. **(B)** The images of the mice liver were recorded, the red arrows indicate the location of partial tumors. **(C)** Mice tumor number and **(D)** liver weight were analyzed. **(E)** Flow cytometry was performed on mouse liver tumors to detect the levels of CD45, F4/80, CD11b, M1 marker CD86 and M2 marker CD206. Macrophages infiltration were analysized by both CD11b^+^ and F4/80^+^ in the tumor tissue. The CD86/CD206 ratio was calculated to assess the polarization status TAMs. *p < 0.05, **p < 0.01, ***p < 0.001.

HCC mouse model was established after DEN/CCl_4_ administration for 20 weeks (Figure 7A). After sacrifice (week 23), all DEN/CCl_4_-treated mice developed HCC with typical histopathological features (data not shown), while no distant metastasis was observed. 1,25(OH)_2_D_3_ (5 μg/kg, s.c.) or suramin (10 mg/kg, i.p.) treatment significantly reduced whitish macroscopic nodules compared to PBS control (*p* < 0.001) (Figures 7B–D), while combination treatment further reduced nodules and hepatic tumor load, with surface nodule count: 21.8 ± 1.69 (PBS) vs. 12.7 ± 0.97 (suramin) vs. 12.3 ± 0.65 (1,25(OH)_2_D_3_) vs. 7.9 ± 0.54 (combination). Flow cytometry analysis revealed that tumor-infiltrating macrophages (CD11b^+^F4/80^+^) in 1,25(OH)_2_D_3_ group significantly increased from 8.13% (PBS) to 33.4% (*p* < 0.01), while significantly decreased in suramin group (21.7%, *p* < 0.05) and combination group (17.95%, *p* < 0.001) (Figure 7E). Additionally, tumor-infiltrating macrophages (CD11b^+^F4/80^+^) showed distinct immunophenotypic changes: M2-like (CD11b^+^F4/80^+^/CD206^+^/CD86^−^) macrophages decreased from 27.07 ± 2.72% (1,25(OH)_2_D_3_) to 15.88 ± 0.78% (combination; *p* < 0.01); and M1-like (CD11b^+^F4/80^+^/CD86^+^/CD206^−^) macrophages increased from 10.51 ± 2.50% (1,25(OH)_2_D_3_) to 25.96 ± 3.72% (combination; *p* < 0.001). Further analysis revealed that CD86^+^/CD206^+^ ratio increased 1.7-fold (*p* < 0.001) with combination therapy versus 1,25(OH)_2_D_3_ alone. These findings demonstrate that suramin enhances 1,25(OH)_2_D_3_ anticancer activity partly via inhibiting monocyte/macrophages infiltration and reprogramming TAMs from protumorigenic M2 to antitumoral M1 phenotype in HCC mouse model.

## 4 Discussion

The recruitment of macrophages to the TME precedes their transformation into TAMs, while the density of TAMs is closely associated with a poor prognosis for solid tumor patients (23). Cathelicidin hCAP18/LL-37 has demonstrated multifaceted tumorigenic properties across malignancies. Beyond its direct oncogenic effects on cancer cells, emerging evidence underscores its immunomodulatory role within the TME. In prostate cancer, overexpressed LL-37 chemo-attracts immature myeloid progenitors to the TME (24). Similarly, murine CRAMP (the functional homolog of LL-37) drives colon cancer progression by recruiting inflammatory cells, particularly macrophages, into the TME (25). Our current investigations reveal that LL-37 overexpression significantly enhances macrophage migration toward HCC cells *in vitro*. This finding aligns with reports demonstrating that supraphysiological concentrations of LL-37 act as potent chemoattractants for phagocytes (monocytes, macrophages, and neutrophils), recruiting them to infection sites either directly or via chemokine induction (26, 27). Notably, such chemotactic gradients may originate not only from infection sites but also from the TME itself, where they persist and create permissive niches for sustained macrophage infiltration into tumors.

Our *in vivo* studies revealed that HCC/macrophage co-xenografts exhibited accelerated growth with enhanced M2 polarization, evidenced by an increased number of CD163^+^/Arg-1^+^ macrophages in tumor. This finding aligns with established mechanisms where HCC cells recruit and trigger M2 polarization through exosomal delivery or by secreting cytokines (IL-4, IL-13, CSF-1) (3, 28). Here we demonstrate that both exogenous LL-37 addition and endogenous overexpression significantly promotiate M2 polarization in THP-1-derived macrophages, whether they were cultured alone or co-cultured with HCC cells. Notably, hCAP18/LL-37 expression showed significant upregulation in M2-type macrophage, HCC/macrophage co-culture, and HCC/macrophages co-xenograft. Establishing a paracrine loop where TAMs themselves become major sources of LL-37. LL-37 upregulationhas similarly been reported to drive M2 polarization in breast, colorectal, and prostate cancer *in vitro* models were reported previously (24, 25, 29). While STAT3 activation has been implicated in LL-37-mediated M2 polarization in prostate cancer (24, 30). Beyond STAT3 activation, our mechanistic investigations uncover a distinct Akt/mTOR-dependent pathway in HCC microenvironment. Phosphorylation analysis revealed that LL-37 stimulation induced 2-fold Akt activation and mTOR phosphorylation, which were completely abrogated by Akt inhibitor MK-2206 and mTOR inhibitor Rapa, respectively. This novel signal provides a mechanistic basis for the enhanced M2-like TAM polarization observed in co-xenograft models, suggesting that LL-37-mediated immunosuppression occurs through parallel STAT3 and Akt/mTOR pathways.

Previous studies have demonstrated that hCAP18/LL-37 attenuates the anticancer efficacy of 1,25(OH)_2_D_3_ in HCC xenograft model (20). Human cathelicidin (hCAP18/LL-37) is directly regulated by vitamin D via the VDR pathway, whereas murine *CRAMP* lacks a functional VDRE in its promoter region (31, 32). To address this research gap, we established humanized co-culture and co-xenograft models using human HCC cells and macrophages. Our findings reveal that 1,25(OH)_2_D_3_ treatment obviously upregulates hCAP18/LL-37 expression in HCC/macrophages co-cultures *in vitro* and in tumor tissues and serum of co-xenografted mice. This observation suggests that clinical vitamin D supplementation might inadvertently elevate LL-37 levels within HCC microenvironments. Given the established protumorigenic effects of LL-37 (24, 25, 29), our results emphasize the necessity for monitoring serum hCAP18/LL-37 dynamics during clinical trials evaluating vitamin D-based therapies for HCC patients.

Vitamin D exhibits dual effects on the immune microenvironment in HCC. While 1,25(OH)_2_D_3_ demonstrates direct anticancer activity through inhibition of proliferation and colony formation, emerging evidence highlights its immunosuppressive potential. Our findings reveal that 1,25(OH)_2_D_3_ promotes M2 polarization (CD163^+^/Arg-1^+^) while suppressing M1 phenotype (iNOS^+^) in *in vitro* co-cultures, *in vivo* co-xenograft and DEN/CCl_4_-induced HCC mouse models, resulting in increased M2: M1 ratio. This immunosuppressive polarization parallels previous observations in inflammatory bowel disease and diabetic nephropathy, where 1,25(OH)_2_D_3_ enhances macrophage M2 skewing (12, 13, 33). Additionally, 1,25(OH)_2_D_3_ treatment (500 nM) also significantly increased macrophage recruitment to HCC cells *in vitro.* Notably, quantitative analysis revealed a significant increase in mouse-derived CD11b^+^macrophage infiltration within 1,25(OH)_2_D_3_-treated tumors compared to PBS control in mouse co-xenograft models (data not shown). Previous study in mammary carcinoma models showed that 1,25(OH)_2_D_3_ may similarly enhance M2 polarization, potentially facilitating metastasis (15). Another study reported that high expression of VDR in pancreatic cancer promotes M2 macrophage polarization and recruitment through the secretion of CCL20, which activates tumor progression (34). Overall, current research on the effect of 1,25(OH)_2_D_3_ on TAMs remains limited, and the claim that 1,25(OH)_2_D_3_ drives macrophage polarization toward an M2-like phenotype remains controversial. Due to the microenvironmental heterogeneity across cancer types, systematic investigations are urgently needed in the future. The immunosuppressive TME induced by 1,25(OH)_2_D_3_ may counteract its therapeutic benefits, as evidenced by increased tumor inhibition rates upon combination with suramin. This observation aligns with Anisiewicz et al.’s proposition that vitamin D supplementation could adversely affect TME dynamics in oncology patients (35), particularly in HCC where LL-37-mediated macrophage activation exacerbates immunosuppression.

Our findings reveal that si-LL-37 significantly enhances 1,25(OH)_2_D_3_’s anticancer efficacy in HCC/macrophage co-cultures. Co-treatment with si-LL-37 potentiated 1,25(OH)_2_D_3_-mediated inhibition of proliferation and colony formation, indicating that LL-37 neutralization reverses 1,25(OH)_2_D_3_’s immunosuppressive effects. Suramin, a clinically approved anti-parasitic agent (36), exhibited dual anticancer mechanisms: (1) direct inhibition of LL-37 function by blocking its membrane binding and cellular uptake in macrophages, and (2) promoting M1 polarization while suppressing M2 polarization and the Akt/mTOR signaling pathway. Notably, suramin completely abolished 1,25(OH)_2_D_3_-driven macrophage recruitment and M2 polarization in co-xenografts, enhancing the tumor inhibition rates from 44% (1,25(OH)_2_D_3_ alone) to 75% (combination therapy), and significantly reducing whitish macroscopic nodules. Although clinical trials showed limited efficacy of vitamin D monotherapy in HCC (10, 37), our discovery of the 1,25(OH)_2_D_3_-LL-37-TAM axis provides a mechanistic explanation for these discrepancies. The paradoxical protumorigenic mechanism of 1,25(OH)_2_D_3_ via LL-37-mediated immunosuppression suggests that combining 1,25(OH)_2_D_3_ with suramin may optimize therapeutic outcomes. These findings warrant clinical evaluation of serum hCAP18/LL-37 as predictive biomarkers and suramin as an adjuvant agent for HCC patients receiving vitamin D-based therapies.

Suramin, a clinically approved agent with a century-long history in trypanosomiasis, has recently gained much more attention for its numerous potential applications (36). Emerging evidence highlights its promising applications in antiviral, antidepressant, and anticancer therapies (38, 39). Our study provides the first demonstration of suramin’s critical role in modulating TAMs and proposes a novel combinatorial strategy with 1,25(OH)_2_D_3_ to enhance therapeutic efficacy in HCC treatment. However, the clinical translation of suramin is hindered by several limitations, including neurotoxicity, poor tissue bioavailability, and poor tissue penetration and retention (40, 41). Though a literature stated that suramin was generally safe and well tolerated in healthy Chinese volunteers at the dose of 10 mg/kg or 15 mg/kg (42). Further pharmacokinetic studies are required to optimize dosing regimens. Furthermore, advanced drug delivery systems should be explored to improve its tumor targeting and minimize off-target effects, thereby accelerating suramin’s clinical application in oncology.

## 5 Conclusion

**Figure 8:**
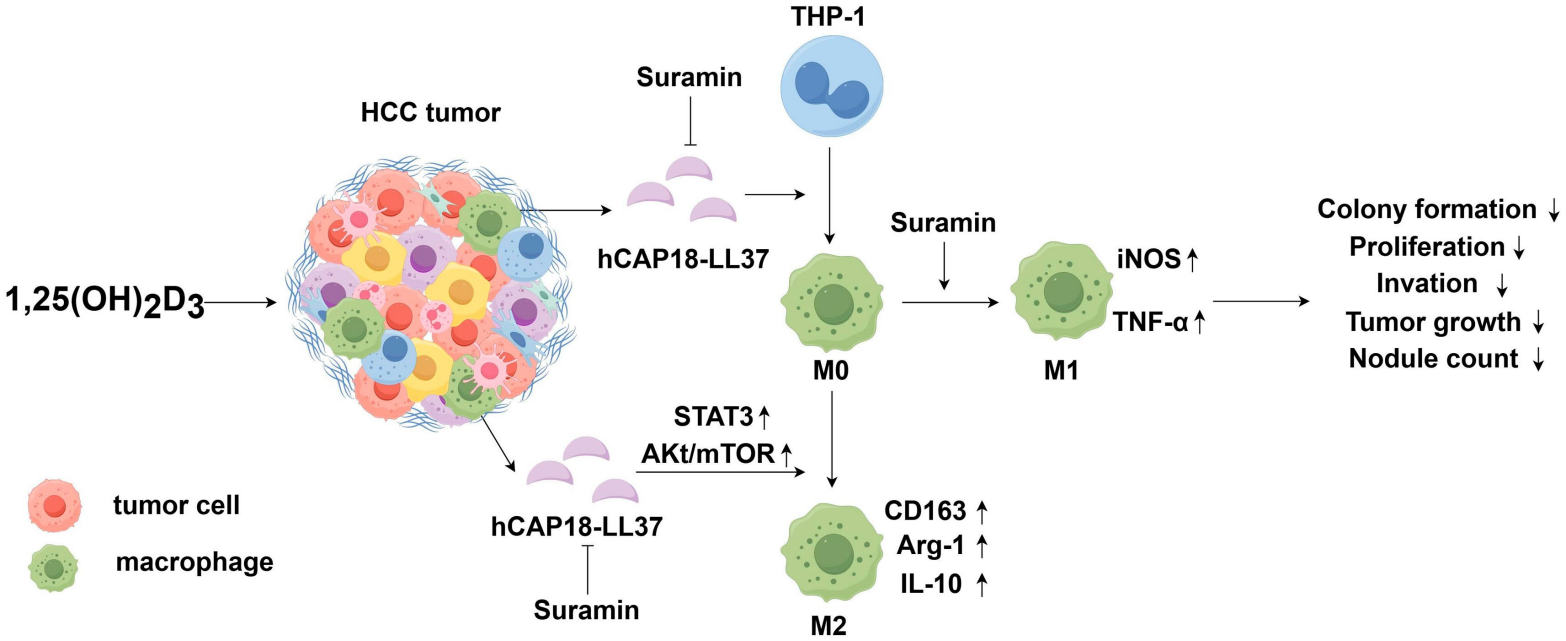
Schematic illustration showing the 1,25(OH)_2_D_3_-LL-37-TAM axis affecting 1,25(OH)_2_D_3_’s anti-cancer efficacy in HCC. 1,25(OH)_2_D_3_ induces robust LL-37 secretion in HCC/macrophages co-cultures. Secreted LL-37 (a) enhances monocyte/macrophage migration toward HCC cells (b) activates AKt/mTOR and STAT3 pathways, and (c) drives M2 polarization, while suppressing M1 phenotype. This LL-37-mediated M2-TAM fosters an immunosuppressive environment that diminishes 1,25(OH)_2_D_3_’s anticancer effects. Suramin inhibits the membrane binding and internalization of LL-37 in macrophages, blocking LL-37-mediated TAMs recruitment and M2 polarization, while promoting antitumor M1 phenotype responses. Suramin-mediated blockade of the 1,25(OH)_2_D_3_-LL-37-TAM axis potentiates 1,25(OH)_2_D_3_’s anticancer efficacy by potentiating the inhibition of HCC cell proliferation, colony formation, invasion, and tumor growth.

This study identifies the 1,25(OH)_2_D_3_-LL-37-TAM axis as a key mechanism underlying the limited efficacy of vitamin D monotherapy in HCC (Figure 8). Specifically, hCAP18/LL-37 acts as a pivotal immunosuppressive mediator, driving monocytes/macrophages recruitment and M2 polarization via 1,25(OH)_2_D_3_-induced transcriptional activation. Mechanistically, LL-37 upregulation by 1,25(OH)_2_D_3_ promotes HCC progression by enhancing proliferation, migration, and invasion through TAM immunosuppressive reprogramming. Importantly, suramin potently antagonizes this axis, blocking LL-37-mediated TAM recruitment and M2 polarization, while promoting antitumor M1 phenotypes. Our results emphasize the necessity for monitoring serum hCAP18/LL-37 dynamics during clinical trials evaluating vitamin D-based therapies for HCC patients. These findings highlight suramin as a promising adjunct to 1,25(OH)_2_D_3_-based immunotherapy for HCC.

## Data Availability

The original contributions presented in the study are included in the article/Supplementary material, further inquiries can be directed to the corresponding author.

## Footnotes

^1^ www.clinicaltrials.gov
