# Supplementary material for: Suramin blocked hCAP18/LL-37-induced macrophage recruitment and M2 polarization to enhance the therapeutic efficacy of 1,25(OH)2D3 against hepatocellular carcinoma in vitro and in vivo mouse model
Source: Front Nutr. 2025 May 16;12:1556533. doi: 10.3389/fnut.2025.1556533 (PMC12122311; doi:10.3389/fnut.2025.1556533)
Supplement: Supplementary file 1 [file Data_Sheet_1.docx]

***Supplementary Material***

**1 Supplementary Tables and Figure**

**1.1 Supplementary Table 1. Primers used in construction of pcDNA/hCAP18 and pcDNA/LL-37 plasmid experiments.**

| Gene Name | Primers | Nucleotide Sequence (5’ to 3’) | Amplified product lengths |
| --- | --- | --- | --- |
| pcDNA/  hCAP18 | F | CGGGATCCATGAAGACCCAAAGGGATGG | 513bp |
|  | R | GCTCTAGA CTAGGACTCTGTCCTGGGTA |  |
| pcDNA/  LL-37 | F | CGGGATCC CTGCTGGGTGATTTCTTCCG | 114bp |
|  | R | GCTCTAGA GGACTCTGTCCTGGGTACAA |  |

**1.2 Table Supplementary Table 2.** **Oligonucleotide sequences of forward and reverse primers for quantitative real-time PCR (qRT-PCR).**

| Gene Name | Forward Primers | Reverse Primers |
| --- | --- | --- |
| LL-37 | CGACACAGCAGTCACCAGAGGA | GAAATCACCCAGCAGGGCAAATC |
| CD163 | GAGGAGGCAGAAGAATGGT | CCACTTCACAGGTGAGGGAC |
| Arg-1 | GTGGAAACTTGCATGGACAAC | CCTGGCACATCGGGAATCTTT |
| iNOS | CATCCTCTTTGCGACAGAGAC | GCAGCTCAGCCTGTACTTATC |
| IL-1β | GCTGATGGCCCTAAACAGATGAA | TCCATGGCCACAACAACTGAC |
| VDR | ACCGTGCCCTGCCTCATTC | TGGTCCCCATGTCTGCCTC |
| *β-*actin | GAGCTACGAGCTGCCTGACG | CCTAGAAGCATTTGCGGTGG |

**1.3 Supplementary Figure
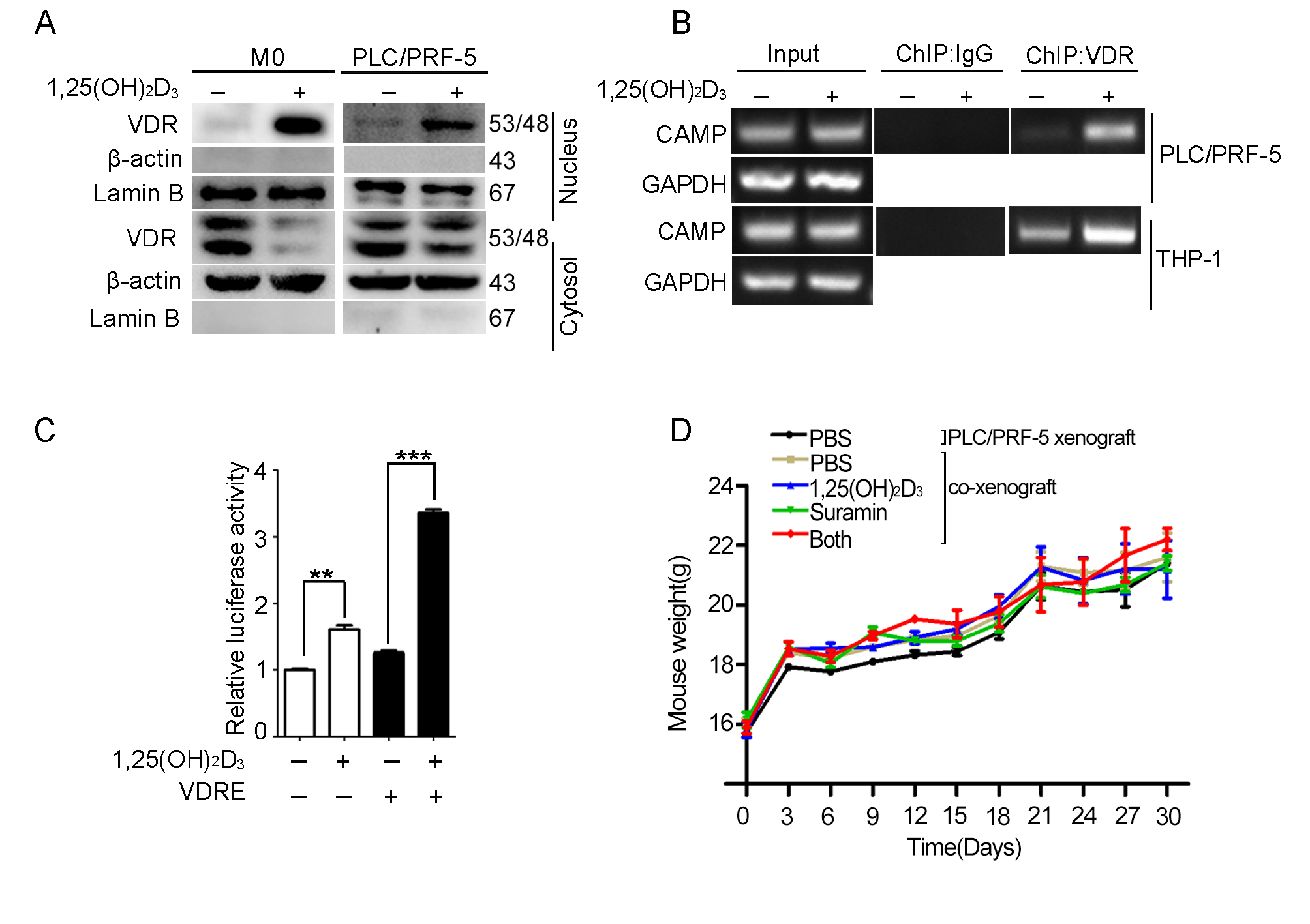
 1**

**Supplementary Figure 1. (A)** M0 and PLC/PRF-5 cells were treated with 1,25(OH)_2_D_3_ for 48 h. Nuclear and cytosolic fractions were isolated from these cells and western blot was conducted to detect the levels of Lamin B1, and VDR and β-actin. **(B)** ChIP assay was conducted to assess the interaction of VDR and the *CAMP* gene promoter in M0 macrophages and PLC/PRF-5 cells, respectively. PCR was performed to amplify the promoter region containing the VDRE. **(C)** Dual luciferase reporter assay was conducted to detect the *CAMP* promoter in macrophages after 1,25(OH)_2_D_3_ treatment. **(D)** Growth curve showing the body weight of mice after different treatments. Four to six weeks-old nude mice were subcutaneously injected with 6 × 10^6^ PLC/PRF-5 cells or a mixture of 6 × 10^6^ PLC/PRF-5 cells plus 1.5 × 10^6^ M0 macrophages to form PLC/PRF-5 xenograft and PLC/PRF-5/macrophages co-xenograft mice models. And then were assigned into PLC/PRF-5 xenograft group and PLC/PRF-5/macrophages co-xenograft groups with different treatment. Difference comparison was conducted between different groups.
